# GLP-1 Receptor Agonists: A New Treatment in Parkinson’s Disease

**DOI:** 10.3390/ijms25073812

**Published:** 2024-03-29

**Authors:** Kallirhoe Kalinderi, Vasileios Papaliagkas, Liana Fidani

**Affiliations:** 1 Laboratory of Medical Biology-Genetics, School of Medicine, Faculty of Health Sciences, Aristotle University of Thessaloniki, 54124 Thessaloniki, Greece; sfidani@auth.gr; 2 Department of Biomedical Sciences, School of Health Sciences, International Hellenic University, 57400 Thessaloniki, Greece; vpapal@auth.gr

**Keywords:** Parkinson’s disease, type 2 diabetes mellitus, GLP1-R agonists, GLP-1, exendin-4, liraglutide, lixisenatide, semaglutide, clinical trials, neurodegeneration

## Abstract

Parkinson’s disease (PD) is one of the most common neurodegenerative diseases. Recent data highlight similarities between neurodegenerative diseases, including PD and type 2 diabetes mellitus (T2DM), suggesting a crucial interplay between the gut–brain axis. Glucagon-like peptide-1 receptor (GLP-1R) agonists, known for their use in T2DM treatment, are currently extensively studied as novel PD modifying agents. For this narrative review article, we searched PubMed and Scopus databases for peer-reviewed research, review articles and clinical trials regarding GLP-1R agonists and PD published in the English language with no time restrictions. We also screened the references of the selected articles for possible additional articles in order to include most of the key recent evidence. Many data on animal models and preclinical studies show that GLP1-R agonists can restore dopamine levels, inhibit dopaminergic loss, attenuate neuronal degeneration and alleviate motor and non-motor features of PD. Evidence from clinical studies is also very promising, enhancing the possibility of adding GLP1-R agonists to the current armamentarium of drugs available for PD treatment.

## 1. Introduction

Parkinson’s disease (PD) is one of the most common neurodegenerative diseases. It affects 1–2% of the population above 65 years, and this percentage rises to 3–5% at ages beyond 85 years [1]. PD prevalence is continually increasing, and by 2040, about 12 million people are expected to be diagnosed with this devastating disease. PD is mainly characterized by motor symptoms such as resting tremor, bradykinesia, rigidity, postural instability and freezing episodes; however, a variety of non-motor features, such as cognitive decline, behavioral symptoms, sleep disturbances, fatigue, autonomic symptoms and sensory problems are also common [2], often as prodromal characteristics of the disease [3,4]. The pathological hallmarks of the disease are the progressive and selective degeneration of the dopaminergic neurons in the substantia nigra, resulting in dopamine depletion in the striatum, and the presence of Lewy bodies in the remaining neurons [1].

Currently, PD treatment aims at the symptomatic relief of PD patients, without being able to prevent or inhibit the process of neurodegeneration. The main target of current PD treatment is the restoration of dopamine levels, as the deficiency of this neurotransmitter is the main cause of PD. However, chronic use of l-dopa, which is the gold standard for current PD treatment, is frequently associated with the development of motor complications, motor fluctuations and dyskinesias, which in the long run cause severe disability in a large percentage of PD patients [4]. Alternative treatment agents are nowadays under intense research, targeting different pathways in PD pathogenesis. Recent data highlight similarities between neurodegenerative diseases, including PD and type 2 diabetes mellitus (T2DM) [5], suggesting that the homeostasis in the gut–brain axis is fundamental for the maintenance of health in both central nervous system (CNS) and peripheral system, and these systems can affect one another in multiple pathways. GLP-1R agonists are licensed by the US Food and Drug Administration (FDA) for the treatment of T2DM; however, these agents are currently extensively studied as novel PD-modifying agents that can have an impact on multiple mechanisms of PD pathology. Interestingly, the prevalence of PD was decreased in T2DM patients who have been prescribed antidiabetic drugs such as GLP-1 receptor agonists or dipeptidyl peptidase IV (DPP-IV) inhibitors, suggesting a possible neuroprotective effect of these agents [6,7]. These drugs have also shown a “stroke protective” effect, highlighting their possible importance in brain disorders [8,9]. A number of initial studies have pinpointed the possible neurotrophic and neuroprotective effects of these agents. GLP-1 agonists have been found to modify amyloid-beta precursor protein processing and protect against oxidative injury, modulate calcium responses to glutamate and membrane depolarization, regulate neuronal plasticity and cell survival and have neuroprotective and neurotrophic properties [10,11,12,13]. This review analyses current data on the common pathogenic pathways between T2DM and PD and describes: (a) the role of glucagon-like peptide-1 (GLP-1) and GLP-1 receptors, (b) the development of GLP-1 receptor agonists and (c) the current data regarding the role of these new drugs as disease-modifying agents based on data from studies on animal models and preclinical as well as clinical studies. The mechanisms of action of GLP-1 receptor agonists as therapeutic targets in PD are also discussed.

## 2. T2DM and PD: Diseases with Overlapping Pathophysiology?

T2DM is a disease characterized by high blood glucose levels due to deficient insulin secretion and insulin resistance of insulin-sensitive tissues such as the liver, adipose tissue and muscles [14]. Importantly, dysregulated insulin signaling has been proposed to be associated with PD pathogenesis either triggering or accelerating the pace of the disease development. More specifically, the bulk of the data supports that T2DM is a risk factor for PD. In a recent systematic review and meta-analysis, T2DM patients had a 1.34 higher risk of developing PD accompanied by more severe motor symptoms [15], whereas previous population-based studies showed that T2DM may increase PD risk by approximately 40% [16,17]. In another study, the risk ratio of PD in patients with T2DM was associated with diabetes duration, increasing to 1.618 in patients with diabetes duration > 5 years [18]. Insulin resistance has also been associated with dopamine degeneration, implicating mainly the AKT insulin signaling [19]. Downstream molecules of this pathway such as forkhead box protein O (FoxO), mechanistic target of rapamycin (mTOR) and glycogen synthase kinase 3β (GSK3β) are implicated in processes such as α-synuclein degradation, mitochondrial biogenesis, oxidative stress and inflammation all being of crucial importance in PD pathogenesis. Interestingly, in diabetes-induced MitoPark mice, insulin resistance has been associated with altered α-synuclein expression, mitochondrial dysfunction and oxidative stress [20]. Moreover, MPTP-mouse models of PD developed insulin resistance, neuroinflammation and increased α-synuclein [21]. Insulin resistance and altered glucose metabolism are highly prevalent in PD patients with dementia [22,23]. Interestingly, in a PD rat model, the activation of the peroxisome proliferator-activated receptor gamma (PPAR-γ), which increases insulin sensitization and glucose metabolism, protected against MPTP-induced memory impairment [24]. Additionally, in a recent 6-OHDA PD rat model study, intranasal insulin attenuated motor deficits and improved mitochondrial function [25]. In a small RCT, intranasal insulin was also associated with improved cognitive function and Unified Parkinson’s Disease Rating Scale (UPDRS) score [26]. However, the long-term use of insulin has been associated with insulin desensitization, decreasing the effectiveness of insulin; thus, new alternative treatments that do not enhance insulin desensitization and do not affect blood glucose levels in normoglycemic patients could overcome these drawbacks in insulin treatment [27]. All the above-mentioned data indicate that T2DM and PD may share some common pathogenic pathways, which have been targeted over the last years for developing therapeutic strategies. In particular, GLP-1R agonists, which have been approved by the FDA for the treatment of T2DM, are recently emerging as promising therapeutic agents in PD, increasing the expectations for identifying new PD drugs that could possibly be neuroprotective or even halt or reverse disease progression.

## 3. GLP-1 and GLP-1 Receptor

GLP-1 is an endogenous 30 amino acid multifunctional peptide produced by proteolytic cleavage of proglucagon molecules and secreted by the distal intestinal ileum and colon L-cells after food intake. GLP-1 is also produced in the CNS at the neuronal level of the solitary tract within the brainstem. GLP-1 has been shown to affect multiple neuronal functions, such as neurogenesis, neurodegeneration, energy homeostasis, thermogenesis, blood pressure control and retinal repair. In the pancreas, GLP-1 enhances insulin secretion and synthesis, promotes pancreatic β-cell proliferation and survival and decreases glucagon release as well as β-pancreatic cell apoptosis. GLP-1 can also increase glucose uptake by muscles and enhance lipolysis and glucose uptake in adipocytes. It can also reduce appetite and slow gut emptying and gastric acid secretion. In the kidneys, it is associated with mild natriuresis. Moreover, GLP-1 increases contractility in the heart and heart rate and has vascular protective effects [28,29]. GLP-1 acts via binding to GLP-1 receptors (GLP1-Rs), which are seven transmembrane-spanning proteins that belong to the class of B1G protein-coupled receptor family. GLP1-Rs consist of 463 amino acids and are expressed in pancreatic islet cells, as well as in other organs, such as the gastrointestinal tract, lung, heart, kidney and brain, exerting indirect metabolic actions [30]. In the brain, it is expressed in the hypothalamus, the hippocampus, the subventricular zone, the striatum, the substantia nigra, the cortex and the brain stem. More specifically, GLP-1R has evidenced expression in neurons, microglia and astrocytes across these key brain regions [31]. GLP-1 has been associated with improved endothelial function and suppression of inflammation, as well as cardioprotection. GLP-1 has a short half-life and is inactivated by the DPP-IV enzyme; however, GLP1-Rs are not cleaved by DPP-IV and can cross the blood–brain barrier (BBB); thus, they are important candidates for treating brain diseases. Overall, GLP-1 and GLP1-Rs are major components of the gut/brain axis and, through the multiple functions that they exert, may be a promising therapeutic target for PD.

## 4. GLP-1 Receptor Agonists

GLP-1 and GLP1-Rs are known as approved agents for T2DM treatment. However, GLP1-Rs, due to their ability to escape inactivation by DPP-IV and to cross the BBB, are promising candidates to treat neurodegenerative diseases such as PD. GLP-1R agonists are divided into short-acting and long-acting, based on the time effect and the volume of injections needed [19,29]. Short-acting preparations, such as exenatide, need to be injected 2–3 times a day, whereas long-acting preparations such as lixisenatide and liraglutide are injected once a day. Long-acting preparations also include drugs such as semaglutide, dulaglutide or a long-acting release formulation of exenatide, which generally need to be injected once a week. Exenatide was the first drug used for T2DM treatment. It is a synthetic version of exendin-4, a peptide that shares 53% amino acid sequence with native GLP-1, is resistant to DPP-IV and binds to GLP-1Rs. Exendin has a short half-life; after a subcutaneous injection > 0.2 μg/kg, it can be detected in the plasma in about 15 min and for approximately 15 h. Lixisenatide is also a synthetic peptide derived from the exendin-4 hormone with an increased half-life of about 3 h and a four-times increased binding affinity with the GLP-1R. It has also a slower rate of dissociation from its receptor, thus a prolonged pharmacological effect. A recent longer-lasting pegylated version of exendin-4 (NLY-01) has also been developed. Liraglutide is a GLP-1 recombinant analog characterized by delayed absorption and extended plasma half-life, over 13 h, due to its binding to albumin. In comparison to short-acting GLP-1R agonists, liraglutide has fewer side effects and a better improvement in lowering glycated hemoglobin as well as fasting blood glucose. Albiglutide is a recombinant fusion protein consisting of two copies of a 30-amino acid sequence of modified human GLP-1, which also binds to albumin and has a half-life of 5 days. Dulaglutide consists of two DPP-IV-protected GLP analogs covalently linked to a human Ig G4-fragment crystallizable (Fc) heavy chain produced via recombinant DNA technology. It has a long half-life period, and the average peak time of subcutaneous injection is 48 h. Semaglutide, another GLP-1 analog, has also reduced susceptibility to DPP-IV. In fact, it is a modification of liraglutide with a much enhanced survival time in the blood. Importantly, dual and triple GLP-1R/Gastric inhibitory polypeptide receptor (GIP-R) agonists have been developed, maximizing the beneficial effects and minimizing the adverse effects of these agents. Among the dual GLP-1R/GIP-R agonists, tirzepatide has a high albumin affinity, thus an increased half-life of 5 days. Structurally, it is composed of a peptide of 39 amino acids with the bioactive sequence of gastric inhibitory polypeptide (GIP) and with a sequence acting on GLP-1, replacing its intermediate amino acid; other agents are DA-JC1, DA2, DA-CH3, DA-JC4 and DA-CH5. Interestingly, monomeric GLP-1R agonists, like semaglutide and liraglutide, as well as dual GLP-1R agonists like tirzepatide, have been FDA-approved for the treatment of T2DM and metabolic disorders, including chronic weight management in adults with obesity [32,33]. A synthetic monomeric peptide triple receptor agonist called triagonist that incorporates GLP-1, GIP and glucagon actions has also been found to have neuroprotective effects [19]. In general, the ability of these drugs to protect the brain is directly associated with their penetration ability in the brain. In a recent study examining the ability of single and dual incretin receptor agonists, exendin-4, lixisenatide, Peptide 17, DA3-CH and DA-JC4 had significant rates of blood-to-brain influx, but liraglutide, semaglutide and Peptide 18 did not measurably cross the BBB. Importantly, among the non-acylated, non-PEGylated incretin receptor agonists that were examined, exendin-4 and DA-JC4 were best able to cross the BBB [34], enhancing their priority as possible therapeutic agents in neurodegenerative diseases, such as PD. In another recent study, it was found that GLP-1R agonists can cross the BBB in a fast and a slow process. In fact, albiglutide and dulaglutide had the fastest brain uptake, followed by DA5-CH, whereas tirzepatide had a slow brain uptake [35]. Importantly, recent evidence from animal models and preclinical and clinical studies support that GLP1-Rs are a new category of molecules that can hopefully open up new avenues in the field of PD treatment.

## 5. GLP-1 Receptor Agonists in Parkinson’s Disease Treatment

Intense research in the field of new therapeutic strategies in PD therapy has highlighted GLP1-R agonists as possible novel therapeutic agents. Studies of animal models of PD as well as preclinical studies show that GLP1-R agonists can restore dopamine levels, inhibit dopaminergic loss, attenuate neuronal degeneration and alleviate motor and non-motor features of PD. Moreover, evidence from clinical studies is also very promising, enhancing the possibility of adding GLP1-R agonists to the current armamentarium of drugs available for PD treatment.

### 5.1. Evidence from Animal Models: Preclinical Studies

Novel incretin analogs including exendin-4 were found to improve autophagy and protect from mitochondrial stress induced by a toxic mitochondrial complex I inhibitor, rotenone in dopaminergic SH-SY5Y neuroblastoma cells, increasing the survival of SH-SY5Y cells [36]. Peripheral administration of GLP-1R agonists increased the expression of tyrosine hydroxylase (TH)-containing neurons [37]. TH is a core enzyme in the pathway of dopamine synthesis [38]. Exenatide was also found to protect dopaminergic neurons from 6-OHDA and MPTP toxicity; increasing dopamine levels and improving motor abilities in diabetic rats with MPTP-induced PD [39,40,41,42]. Moreover, continuous exendin-4 administration had a protective effect on cognitive-related neurotransmission systems and decreased the death of hippocampal neurons induced by injection of toxin lipopolysaccharide in mice [43]. In a Parkinsonian rat model of α-synucleinopathy, exendin-4 alleviated TH-positive neuronal loss and terminal denervation, affected the expression of a functional component of monoaminergic neurotransmission, vesicular monoamine transporter 2, in the nigrostriatal dopaminergic systems of rats and improved motor symptoms [44]. In another mouse model, a modified form of exenatide (NLY01) protected against the loss of dopaminergic neurons through the direct prevention of microglial-mediated conversion of astrocytes to a neurotoxic phenotype [45]. The anti-inflammatory effects of GLP-1 receptor agonists have also been found to be mediated via inhibiting Toll-like receptor agonist-induced inflammation [46]. Interestingly, in a comparison dual agonist DA5-CH and NLY01 MPTP mouse model study, the dual agonist was found to be more effective compared to NLY01 regarding PD pathology [47]. A sustained-release exenatide agent, PT302, was found to sustain dopaminergic neurons after 6-OHDA lesioning in rats [48]. Interestingly, in a recent study in a PD mouse model, early treatment with PT320 ameliorated L-DOPA-induced dyskinesia, highlighting its possible ability to mitigate dopaminergic degeneration [49]. Notably, in another study, lixisenatide and liraglutide were found to be more effective regarding protection against MPTP-induced dopaminergic degeneration compared to exenatide [50]; however, these results need to be replicated [51]. In a recent MPTP PD mouse study, both exendin-4 and linagliptin reversed motor dysfunction, glial activation and dopaminergic neuronal death [52]. Importantly, the once-weekly administration of semaglutide was more efficient compared to once-daily liraglutide in restoring TH levels in MPTP-treated mice [53,54]. Neuroprotective effects were also observed for a novel GLP-1 analog with a longer serum half-life than exendin-4, Val(8)GLP-1-GluPal in a mouse MPTP PD model [55]. Additionally, in a rotenone model of PD, sitagliptin and liraglutide improved motor performance and reversed rotenone-induced nigral neuronal loss, inhibiting the inflammatory–apoptotic degenerative process [56,57]. 

Regarding dual GLP-1/GIP receptor agonists, DA-JC1 has been observed to have a neuroprotective effect in an MPTP mouse model [58,59,60], as well as in a cell culture experiment, in SH-SY5Y cells with ROT-induced mitochondrial stress [35], which was also superior to older GLP-1 analogs [61]. In another MPTP mouse study, DA3-CH was better compared to liraglutide in rescuing TH levels [62]. In a recent study, DA5-CH was more effective compared to semaglutide regarding the protection of dopaminergic neurons, the suppression of inflammation and the increase in TH expression in the substantia nigra. Aggregation of α-synuclein was reduced by both drugs, as well as insulin resistance with DA5-CH displaying better results [63]. In ROT-lesioned rats, the dual GLP-1R/GIPR agonist could improve the motor symptoms of PD too [64]. In a comparison MPTP PD mouse model study comparing liraglutide with DA-JC1, DA-JC4 and DA-CH5 at the same dose, DA-JC4 and DA-CH5 were the most effective [65,66]. Furthermore, DA-CH5 was found to be more effective compared to NLY01 as well, in suppressing neurodegeneration and inflammation [47,67]. 

### 5.2. Evidence from Clinical Studies

A number of clinical studies have already been conducted regarding GLP1R agonists and PD, and the initial results are very promising regarding the effects of these drugs in the improvement of PD pathology (Table 1).

In a randomized, single-blind, open-label trial (NCT01174810) [68], exendin-4 was administered twice a day in 45 PD patients with moderate disease for 1 year. The patients were on conventional PD treatment. PD patients not taking this agent were considered as controls. In this study, there was a 2-month wash-out period. After 14 months, there was a significant difference in motor and cognitive function measured by the Movement Disorders Society Unified PD Rating Scale (MDS-UPDRS) and Mattis dementia rating scale-2 (Mattis DRS-2), respectively. These beneficial results were sustained in the follow-up assessment after a wash-out phase of 12 weeks [72]. There were no significant changes regarding depression or subjective ratings of quality of life. The untreated control group rapidly deteriorated in the same time period, highlighting the disease-modifying properties of exendin-4. Regarding adverse events reported by trial participants, weight loss was an important concern and prevented trial completion in one individual; however, this effect was fully reversed after drug discontinuation. Gastrointestinal symptoms, mostly constipation and nausea, were also common side effects of exenatide. None of the serious adverse events observed were considered to be reactions to exenatide. The most common side effects reported in the group of conventional PD medication were constipation, increased “off-medication” time and weight gain. Interestingly, there was a greater increase in the mean dyskinesia rating scale score in the exenatide-treated group, which necessitated a reduction in l-dopa doses in five patients. 

Due to these positive results, a randomized, double-blind, placebo-controlled phase II clinical study (NCT01971242) [69], was conducted by the same research team with subcutaneous administration of exendin-4, one injection/week for 48 weeks in 60 PD patients with moderate disease. The patients were on conventional PD treatment. In this study, there was a wash-out period of 12 weeks. The PD patients that were administered exendin-4 had better motor control in comparison with the placebo group after 48 weeks of drug therapy, and these results were retained after 60 weeks. This persisting beneficial effect of exenatide could suggest that this drug may have a longer-lasting impact on disease severity compared to conventional PD drugs. The frequency of adverse effects including gastrointestinal symptoms did not differ significantly between studied groups. Eight serious adverse events, six in the exenatide and two in the placebo group, were recorded; however, none were judged to be related to the study interventions. Three patients discontinued this study, one in the exenatide group due to asymptomatic hyperamylasemia at 12 weeks and two in the placebo group due to worsening anxiety and dyskinesia, respectively. Moreover, one patient in the placebo group developed pancreatic cancer shortly after the end of the trial monitoring period. A post hoc analysis showed that non-motor signs, such as ‘emotional well-being’ and mood/apathy scores, were also better in PD patients on exenatide treatment, but these results were not sustained after discontinuation of therapy [73]. An additional post hoc analysis also showed that obese PD patients with insulin resistance may have better cognitive results with exenatide in comparison to other PD subgroups [74]. Of note, neuronal-derived exosomes from patients who participated in this phase II trial had higher levels of insulin receptor substrate 1 (IRS-1) phosphorylation at tyrosine sites and higher levels of phosphorylated mTOR and phosphoinositide-3-kinase/Akt (PI3K/AKT) expression in PD patients treated with exenatide compared to the placebo group [75]. 

A randomized, double-blind, phase II clinical trial was also conducted regarding the effect of liraglutide in PD patients (NCT02953665). In this study, 37 active and 18 placebo subjects were included. Subcutaneous injections of liraglutide were administered for 52 weeks in PD patients who were on conventional PD treatment. This study documented significant improvement in the daily living of PD patients who were on liraglutide treatment [70]. Injection site reactions and gastrointestinal symptoms were common adverse effects. Eleven serious adverse effects were reported, but none were related to the study intervention. Further parameters are still being analyzed.

In another randomized, double-blind, placebo-controlled clinical trial (NCT03439943), lixisenatide was administered daily for 1 year in patients with early PD. Patients were examined both during OFF and ON times. PD patients on lixisenatide treatment showed less disability on the Movement Disorder Society-Unified Parkinson’s Disease Rating Scale (MDS-UPDRS III), and this improvement was eminent in ON and OFF times [71]. Nausea was a common side effect in the lixisenatide group.

Other clinical trials examining the effect of exendin-4 (NCT04232969, NCT03456687, NCT04305002), PT320 (NCT04269642), semaglutide (NCT03659682) and NLY01 (NCT04154072) in PD patients are also ongoing, and results are awaited (Table 2).

Interestingly, current data show variability in response to GLP-1R agonists in different PD models and patient populations. Several factors, such as genetic alterations, the stage of the disease or PD comorbidities, can influence the effectiveness of GLP-1R agonists. These parameters may also affect the occurrence of side effects during the treatment with GLP-1R agonists. A personalized treatment approach would aid the early prediction of treatment response and appropriate selection of patients optimizing therapeutic effects and safety. Systematic studies that would address these interindividual differences in treatment response are expected to facilitate individualized treatment.

### 5.3. Mechanism of Action

**Figure 1:**
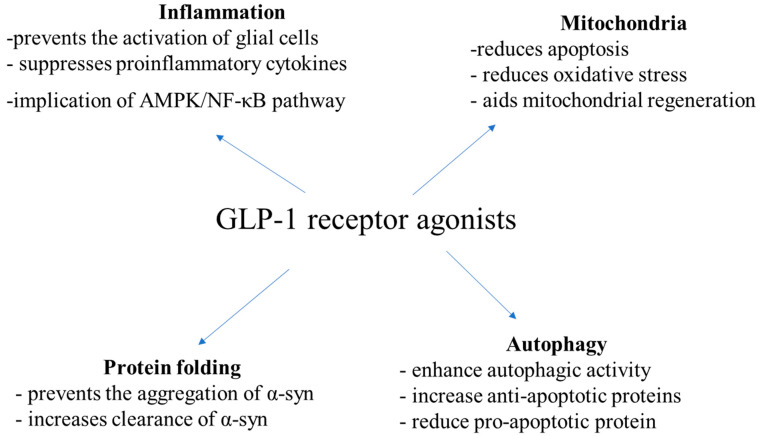
Mechanisms of action of GLP1 receptor agonists according to in vitro and in vivo PD studies.

PD is a heterogenous neurodegenerative disease with a complex etiology. Multiple pathways have been implicated in PD pathogenesis, such as protein misfolding and aggregation, defects in the ubiquitin–proteasome system and aggregation, inflammation, impaired oxidative stress and mitochondrial dysfunction. Current evidence indicates that GLP-1Rs affect multiple of these pathways (Figure 1). 

In in vitro and in vivo studies, exendin-4 prevented the activation of glial cells, restricting neuroinflammation and neurodegeneration [52,76,77,78]. Also, GLP-1 had anti-inflammatory effects in models with LPS-induced lesions [76,79]. Moreover, in a Parkinsonian rat model of α-synucleinopathy, exenatide-4 decreased levels of TNF-α and IL-1β in a dose-dependent way [80], as well as in lesions induced by 6-OHDA [61] and ROT [41]. In other studies, exendin-4 was found to decrease IL-6 levels, as well as NF-kB and cyclooxygenase1 (COX1), all of which are important mediators of inflammation [42,80]. Liraglutide and sitagliptin have been observed to decrease microglial activation and inflammation in ROH-treated rats, too [56,57]. In a mouse MPTP model of PD, liraglutide has been suggested to exert its neuroprotective effects against inflammation via the AMP-activated protein kinase (AMPK)/NF-κB pathway [81]. Novel GLP-1/GIP receptor dual agonists have also been shown to affect microglia activation and levels of pro-inflammatory cytokines [66,67].

Except for inflammation, GLP-1Rs have been found to affect oxidative stress and mitochondrial homeostasis, as well. In rodents with 6-OHDA and ROT-induced lesions, GLP-1R was associated with increased expression of B-cell lymphoma-2 (Bcl-2) and complex I and reduced expression of caspase-3, halting cell death [82,83]. In 6-OHDA-treated SH-SY5Y cells, exendin-4 and DA-CH5 reduced ROS levels, too [61]. GLP-1 has been suggested to decrease oxidative stress via receptor-mediated stimulation of the cyclic AMP, PI3K and protein kinase C pathways and activation of nuclear factor erythroid 2-related factor 2 (Nrf-2) [84]. Furthermore, in mice treated with MPTP, liraglutide has been observed to increase peroxisome proliferator-activated receptor-γ coactivator-1α (PGC-1α) levels and increase NRF2 expression, aiding mitochondrial regeneration [85].

Regarding the implication of GLP-1RA in the process of protein folding, liraglutide has been shown to decrease MPTP-induced α-syn aggregation in mice [86]. Exendin-4 was also associated with increased clearance of total α-syn and pathological pSer129-α-syn via autophagy in the substantia nigra pars compacta of rats, probably via suppression of the PI3K/Akt/mTOR signaling pathway [44]. NLY01 has been shown to affect the aggregation of α-syn in dopamine neurons, too [45]. 

DA-CH5 and exendin-4 have been shown to increase the expression of sequestosome-1 and Beclin-1, affecting also the process of autophagy [61]. Exendin-4 has been observed to increase anti-apoptotic proteins, phospho-Bcl-2 (Ser70) and phospho-BAD (Ser112), and reduce the pro-apoptotic protein Bax and inhibit caspase-3 activity, as well [35,87]. Notably, in rat 6-OHDA models, DA-CH5 and exendin-4 have been suggested to exert their neuroprotective by inducing autophagy and inhibiting apoptosis, particularly by enhancing autophagic activity and reducing the Bax/Bcl-2 and active caspase-3/caspase-3 ratios [35]. Downregulation of apoptotic pathways, including poly (adenosine diphosphate (ADP) ribose) polymerase (PARP) and activation of the PI3K/Akt signaling pathway has also been observed [88]. GLP-1 mimetics have also been found to increase autophagy by promoting the expression of autophagy-related 3, autophagy-related 7 and LC3A/B in SH-SY5Y cells with ROT-induced mitochondrial damage [89]. In AAV-A53T-α-rats, exendin-4 also enhanced autophagy, increasing the expression of LC3-II and down-regulating mTOR and Akt [43]. In a mice MPTP model, liraglutide affected levels of fission and fusion mitochondrial proteins, restoring mitochondrial morphology [85,86]. In a recent study in SH-SY5Y cells treated with 6-OHDA, both semaglutide and liraglutide protected against 6-OHDA cytotoxicity by increasing autophagy flux and decreasing oxidative stress as well as mitochondrial dysfunction, with semaglutide being superior compared to liraglutide [90].

## 6. Research Gaps and Future Directions

Current data have shown that there is a clear correlation between the ability of GLP-1R agonists to cross the BBB and their neuroprotective ability. Significant pharmacodynamic and pharmacokinetic differences exist between different drug classes and compounds of the same class, closely associated with their efficacy. In clinical practice, some patients may not respond to GLP-1R agonists as expected, and systematic studies that would address these interindividual differences in treatment response are still lacking. The optimal interpretation of data may be hampered due to the small sample size or high study heterogeneity. Thus, larger clinical trials examining different GLP-1 analogs in PD are needed in order to improve our knowledge regarding their efficacy, optimal dosing and long-term safety profiles. Hopefully, if validated in future clinical studies, genotyping for GLP1R variants could support the early prediction of treatment response and selection of patients for whom treatment with GLP-1R agonists would have maximum clinical effectiveness and safety. Future research should further explore the underlying mechanisms through which these agents exert their neuroprotective effects, as well. Identifying crucial pathways to target in PD treatment is awaited to improve treatment outcomes. Large-scale clinical trials in diverse PD populations assessing long-term outcomes are also essential, to draw more definite conclusions. The inclusion of a washout period in clinical trial design or long-term follow-up is essential to confirm that any differences in clinical outcome measures observed between treatment groups are clear evidence of disease modification rather than symptomatic effects. Assessment of different biomarkers would also enhance evidence of disease modification. Additional RCTs are also needed in order to examine in detail drug effects on both motor and non-motor symptoms. Personalized treatment approaches according to specific phenotyping and genotyping data are awaited to increase therapeutic benefits. Artificial intelligence is expected to facilitate close monitoring of PD patients’ symptomatology and promote individualized treatment. Precision medicine approaches and the exploration of combination therapies are promising in improving treatment outcomes in PD. Novel dual GLP-1/GIP receptor agonists that can cross the BBB at an enhanced rate should also be examined. Improving drug delivery across the BBB could be another important direction. More friendly routes of administration, e.g., oral forms, are also expected to be examined. The possible interaction between GLP-1R agonists and conventional dopaminergic drugs must also be evaluated in future study designs. The potential impact of these drugs on other neurodegenerative disorders would also be of great value. Interestingly, PD appears to be a multi-organ disorder with a complex interplay between peripheral and central organs. The gut–brain axis emerges as being of great importance, and further research is needed to clarify whether insulin resistance is a cause or consequence of neurodegeneration in PD. Additional information on this issue may aid identification of high-risk individuals, for instance, those with metabolic syndrome, tailoring individualized therapy. Future research focusing on the development of nonpeptidergic ligands that could possibly modify the activity of the GLP-1R itself, maximizing the potent effects of GLP-1R agonists at multiple levels, could possibly offer greater benefits in the treatment of PD, as well as of other neurodegenerative diseases.

## 7. Conclusions

PD is a the second most common neurodegenerative disease. Treatment is mainly symptomatic and consists of dopamine replacement therapy. Interestingly, recent data suggest that dysregulated insulin signaling may be associated with PD, insulin resistance may be implicated in dopamine degeneration and altered glucose metabolism is present in multiple brain regions of PD patients. In line with these data, T2DM has been observed to be a risk factor for PD. Interestingly, GLP-1R agonists, which have been approved by the FDA for the treatment of T2DM, are recently emerging as promising, therapeutic agents in PD. Many data on animal models and preclinical studies show that GLP1-R agonists can restore dopamine levels, inhibit dopaminergic loss, attenuate neuronal degeneration and alleviate motor and non-motor features of PD. Clinical studies regarding the role of GLP1-R agonists, such as exendin-4, liraglutide and lixisenatide in PD also show improvement in motor and cognitive functions, as well as in the daily living parameters of these patients, reinforcing the ambition to identify novel PD modifying agents. GLP1-R agonists have been proposed to affect neuroinflammation, oxidative stress, mitochondrial homeostasis, protein folding, autophagy and apoptosis, pathways that have already been implicated in PD pathogenesis. The results of several clinical trials are also awaited to be available soon, aiming to achieve the challenging but also feasible goal of halting or even reversing progression of this devastating disease.

## Figures and Tables

**Table 1:** Results of GLP-1Rs agonist clinical trials in PD patients.

ClinicalTrial.Gov Identifier	Drug	Result	Reference
NCT01174810	Exendin-4	Improvement in MDS-UPDRS and Mattis DRS-2	[68]
NCT01971242	Exendin-4	Improvement in MDS-UPDRS	[69]
NCT02953665	Liraglutide	Improvement in daily living of PD patients	[70]
NCT03439943	Lixisenatide	Improvement in MDS-UPDRS III	[71]

**Table 2:** Ongoing GLP-1R agonist clinical trials in PD patients.

Title	ClinicalTrials.gov ID	Phase	Status	Intervention	Results Overview
Exenatide Once Weekly Over 2 Years as a Potential Disease Modifying Treatment for Parkinson’s Disease (Exenatide-PD3)	NCT04232969	Phase 3	Active, not recruiting	Drug: Exenatide extended release 2 mg (Bydureon)	Result not yet available
Effects of Exenatide on Motor Function and the Brain	NCT03456687	Phase 1	Completed	Drug: Exenatide	No result available
Exenatide Treatment in Parkinson’s Disease	NCT04305002	Phase 2	Active, not recruiting	Drug: Exenatide Other: Placebo	No result available
SR-Exenatide (PT320) to Evaluate Efficacy and Safety in Patients With Early Parkinson’s Disease	NCT04269642	Phase 2	Active, not recruiting	Drug: PT320 2.0 mg Placebo Drug: PT320 2.0 mg Drug: PT320 2.5 mg	Unknown status
GLP1R in Parkinson’s Disease (GIPD)	NCT03659682	Phase 2	Not yet recruiting	Drug: Semaglutide	No result available
A Clinical Study of NLY01 in Patient’s With Early Parkinson’s Disease	NCT04154072	Phase 2	Active, not recruiting	Drug: NLY01 Drug: Vehicle	No result available

## Data Availability

No new data were created or analyzed in this study. Data sharing is not applicable to this article.
